# Necrotizing Enterocolitis Due to Mesenteric Artery Thrombosis in a Patient with Craniofrontonasal Dysplasia: Casual or Causal Association?

**DOI:** 10.3390/jcm14197055

**Published:** 2025-10-06

**Authors:** Gregorio Serra, Deborah Bacile, Maria Rita Di Pace, Alessandra Giliberti, Mario Giuffré, Marco Pensabene, Giusy Ranucci, Maria Sergio, Giovanni Corsello, Rosaria Nardello

**Affiliations:** 1 Department of Health Promotion, Mother and Child Care, Internal Medicine and Medical Specialties “G. D’Alessandro”, University of Palermo, 90127 Palermo, Italy; baciledeborah@gmail.com (D.B.); mariarita.dipace@unipa.it (M.R.D.P.); gilale98@gmail.com (A.G.); mario.giuffre@unipa.it (M.G.); marco.pensabene@policlinico.pa.it (M.P.); maria.sergio@unipa.it (M.S.); giovanni.corsello@unipa.it (G.C.); rosaria.nardello@unipa.it (R.N.); 2 Pediatric Department for the Treatment and Study of Abdominal Diseases and Abdominal Transplantation, IRCCS ISMETT (Mediterranean Institute for Transplantation and Advanced Specialized Therapies), University of Pittsburgh Medical Center, 90127 Palermo, Italy; granucci@ismett.edu

**Keywords:** CFND, *EFNB1* gene, newborn, thrombosis, NEC, surgery, case report

## Abstract

**Background**: Craniofrontonasal dysplasia (CFND) is an X-linked developmental disorder caused by mutations in the *EFNB1* gene located on chromosome Xq13. This gene encodes ephrin-B1, a ligand for Eph receptors, which is involved in cell signaling pathways and the development of the nervous and vascular systems, as well as facial and cranial structures. Paradoxically, the syndrome manifests with greater severity in heterozygous females, whereas hemizygous males typically present with mild or no abnormalities. **Methods and Results**: We report the case of a late preterm female neonate with dysmorphic features at birth, who subsequently developed necrotizing enterocolitis (NEC) caused by thrombosis of the superior mesenteric artery. Extensive bowel resection led to short bowel syndrome, resulting in cholestatic liver disease, malabsorption, and growth impairment. Array-comparative genomic hybridization (a-CGH) analysis identified a ~791 Kb microduplication at Xq13.1, encompassing the *EFNB1* gene, confirming the diagnosis of CFND. She was enrolled in a multidisciplinary follow-up program and, at 2 years of age, presents with marked growth and neurodevelopmental delay. **Conclusions**: This report describes a rare association between CFND and NEC caused by superior mesenteric artery thrombosis. To the best of our knowledge, no previously reported cases of CFND associated with thrombosis or thrombosis-related conditions, including NEC, have been identified. This is based on a literature review (2004–2025) performed using PubMed and Scopus, and limited to English-language case reports and reviews.

## 1. Introduction

Craniofrontonasal dysplasia (CFND) was first recognized in 1979 by Cohen, who introduced the term to describe a subset of frontonasal dysplasia [1]. CFND is a rare inherited disorder with an estimated incidence of 1:100,000–1:120,000. It is an X-linked craniofacial condition with an unusual pattern of manifestation. Affected females typically present with a range of craniofacial malformations, including hypertelorism, a bifid nose with two separate nostrils, craniosynostosis, other skeletal anomalies, and cognitive delay. Conversely, hemizygous males exhibit a milder phenotype, often with only subtle or no clinical abnormalities. Mutations in the *EFNB1* gene, which encodes ephrin-B1, are detected in the majority of both familial and sporadic CFND cases. *EFNB1* is located on chromosome Xq13.1 and encodes a transmembrane protein involved in bidirectional cell signaling [2], which plays a crucial role in the development of the frontonasal neural crest [3]. Random X-chromosome inactivation leads to functional mosaicism in heterozygous females, explaining their more severe phenotypic presentation. We report a rare case of a late-preterm female newborn with CFND due to a 791 Kb microduplication on chromosome Xq13.1, encompassing *EFNB1*. The patient developed necrotizing enterocolitis (NEC) secondary to superior mesenteric artery thrombosis, which led to short bowel syndrome, resulting in malabsorption, cholestatic liver disease, and ultimately growth retardation.

## 2. Case Report

**Figure 1:**
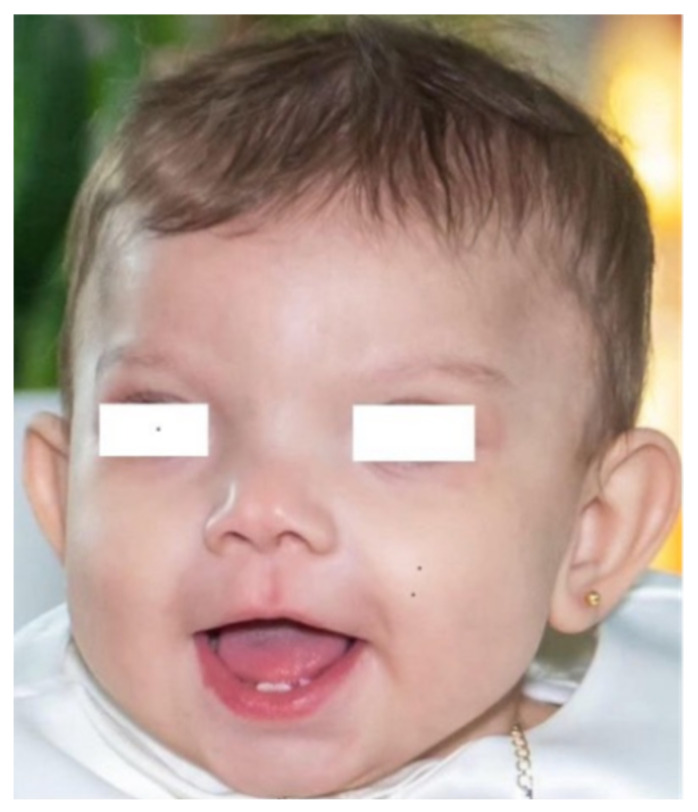
Our patient at age 6 months: notable features include plagiocephaly and craniofacial asymmetry, frizzy hair, prominent frontal drafts, wide and posteriorly rotated ears, medially sparse eyebrows, broad and bifid tip of the nose, short and thick philtrum, thin upper lip.

A female late preterm neonate was born at 36 + 4 weeks of gestation by emergency cesarean section, due to flowmetric placental/fetal alterations (abnormally high umbilical pulsatility index [PI, >95th percentile], associated with a significantly low PI [<3rd percentile] of the fetal middle cerebral arteries). She was the first child of non-consanguineous parents. The mother was affected by type 1 diabetes mellitus, poorly controlled by insulin treatment (glycosylated hemoglobin, HbA1C, >7%). The adaptation to extrauterine life was regular, and Apgar scores were 9 and 10, respectively, at 1 and 5 min. At birth, anthropometric measures were as follows: weight 3140 g (81st percentile, +0.89 standard deviations, SD), length 51 cm (95th percentile, +1.65 SD), occipitofrontal circumference (OFC) 33 cm (41st percentile, −0.23 SD), according with the Italian Ines Growth Charts [4]. Physical examination disclosed dysmorphic features, including plagiocephaly and craniofacial asymmetry, prominent frontal drafts, wide and posteriorly rotated ears, epicanthus, hypertelorism, broad and bifid tip of the nose, short and thick philtrum, thin upper lip, high-arched palate (Figure 1).

**Figure 2:**
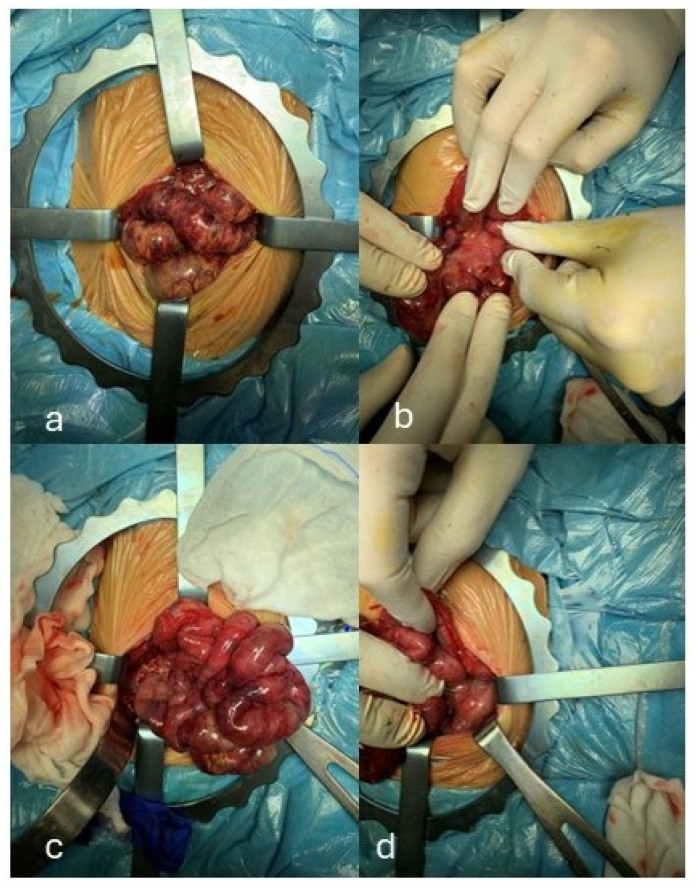
(**a**–**d**). Intraoperative view. (**a**) Entry into the abdominal cavity: approximately 10 cm distal to the ligament of Treitz, a compromised bowel loop with necrotic, friable areas is observed. (**b**) Assessment of the mesenteric root showing a normal ligament of Treitz, without evidence of malrotation or volvulus. (**c**) Proximal jejunal loops with adequate perfusion. (**d**) Adequate perfusion of the descending colon.

Joint laxity, sloping shoulder blades, hypoplasia of the pectoral muscle, *pectus excavatum*, brachydactyly of hands and feet, and widening of the first toe and clinodactyly of the fifth one, bilaterally, were also observed. Postnatally, she manifested mild respiratory distress, which required non-invasive ventilatory support for the first hours of life. A week after birth, abdominal protrusion along with *livedo reticularis* at lower limbs, decreased skin perfusion (capillary refill time of 3 s), and deterioration of the general conditions were noted. Blood tests have been, then, performed, revealing anemia (hemoglobin 8.8 g/dL), thrombocytopenia (PLT: 32,000/μL), in addition to increased levels of inflammation markers (C-reactive protein, CRP, 22.9 mg/L, normal values [n.v.] < 5; interleukin-6, IL-6, 5000 pg/mL, n.v. < 0.8) and lactic dehydrogenase (>2500 IU/L, n.v. 50–250), as well as cholestasis (direct bilirubin 3.88 mg/dL; indirect bilirubin 6.12 mg/dL) and alteration of the coagulative profile (D-dimer 19742 ng/mL, n.v. 0–500; functional antithrombin 51%, n.v. 80–120). A picture of disseminated intravascular coagulation syndrome was, then, suspected. Cerebral ultrasound (US) showed mild periventricular hyperechogenicity, while echocardiography documented wide *foramen ovale* as well as tricuspid insufficiency. Abdominal US showed fluid effusion among the intestinal loops, which further supported the clinically based decision to carry out an X-ray. It evidenced multiple air-fluid levels within the central abdominal bowel tracts, according with small intestine obstruction. An exploratory laparotomy was therefore performed, resulting in the creation of a jejunostomy, with the bowel stumps located approximately 8 cm from the ligament of Treitz. Intraoperative findings highlighted a macroscopic picture compatible with NEC, as well as signs suggestive of thrombosis of the superior mesenteric artery (Figure 2a–d). Anatomopathological analysis, indeed, revealed severe chronic active inflammation of the mucosa of the small intestine, which appeared ulcerated, associated with widespread congestion and vascular ectasia, blood extravasation, granulation tissue and areas of abscess formation.

**Figure 3:**
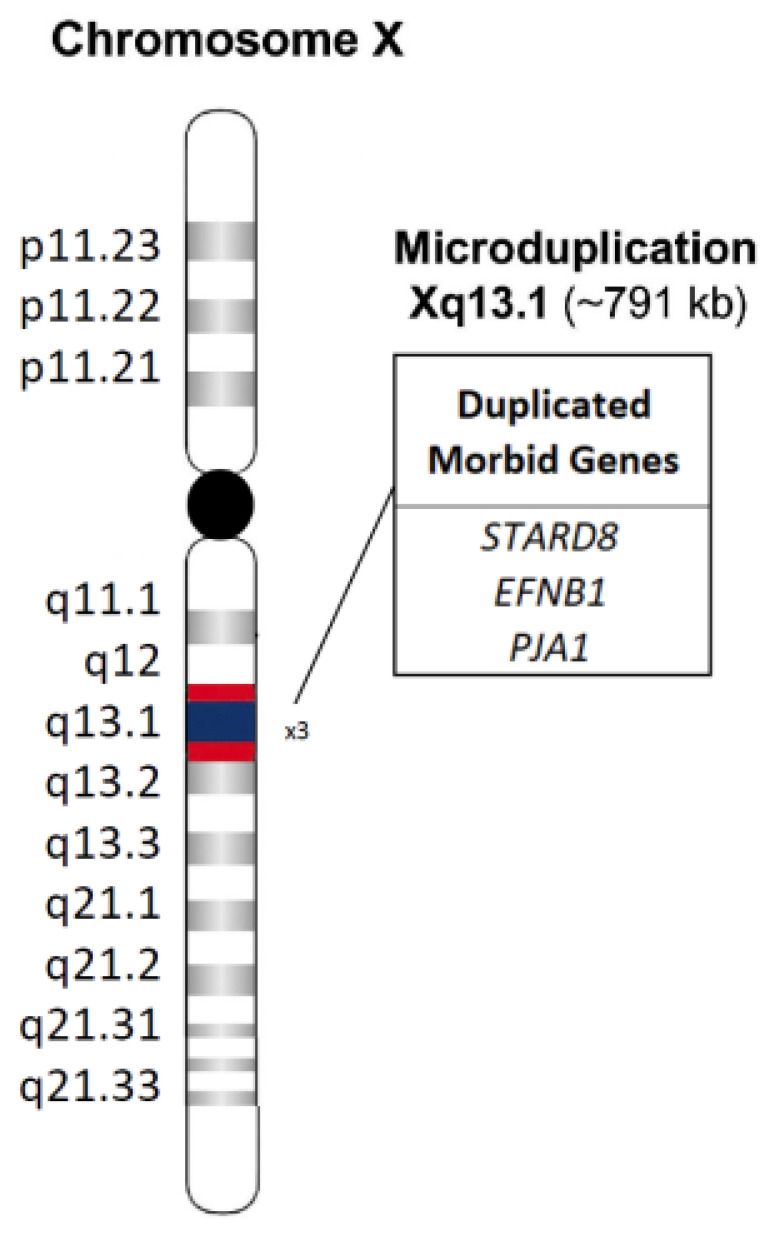
Overview of the Xq13.1 region and its gene content, showing present patient’s microduplication spanning about 791 Kb of genomic DNA, from position 68,048,864 to 68,062,003, according to DECIPHER Genome Browser (GRCh37/hg19 assembly) [5].

In consideration of the dysmorphic features, a molecular cytogenetic analysis through array-comparative genomic hybridization (a-CGH) was performed. The test was carried out with the Agilent GenetiSure Postnatal Research CGH+SNP Microarray (Agilent Technologies^®^, Santa Clara, CA, USA), analyzed using Agilent Cytogenomics software (version 5.0.2.25). The array was 2 × 400 K, with a mean resolution of 9.5 Kb, exon coverage of 89% according to the International Standard Cytogenomic Array (ISCA) guidelines, and ≥3 probes per exon. It documented a microduplication of 791 Kb on chromosome Xq13.1 (GRCh37, nucleotide positions 68,048,864–68,062,003), including the OMIM genes *EFNB1*, *STARD8* (StAR related lipid transfer domain containing 8) and *PJA1* (praja ring finger ubiquitin ligase 1), for diagnosis of craniofrontonasal dysplasia (Figure 3). Such genetic investigation, later extended to the parents, identified the same genomic rearrangement in the father, who conversely showed isolated hypertelorism.

A genetic screening for thrombophilia (*MTHFR*, *PAI-1*, Leiden Factor II and V) was also performed and revealed the A1298C polymorphism of the *MTHFR* gene in homozygosity. Other possible causes of liver damage underlying the thrombotic event were excluded based on specific tests, including alpha-1 antitrypsin levels, serum copper (cupremia), and a comprehensive infectious screening for major and minor hepatotropic viruses, as well as other relevant microorganisms. Plasma levels of protein C (125%, n.v. 70–140), protein S (122%, n.v. 60–123) and antithrombin (30,5%, n.v. 20–40) were measured in the proband and her parents, revealing normal values. In addition, antiphospholipid antibodies were tested in the mother and found to be absent (anti beta 2-glycoprotein IgG/IgM: 8.4/7.1 IU/mL, n.v. for both immunoglobulin classes < 20, anti-cardiolipin IgA/IgG: 4.6/11.4 IU/mL, n.v. for both <20, lupus anticoagulant ratio: 0.9, n.v. < 1.2). Subsequently, due to the occurrence of late-onset sepsis—confirmed by a blood culture positive for *Klebsiella pneumoniae*—complicated by thrombocytopenia (PLT: 19,000/μL), the patient required platelet transfusions and antibiotic therapy (amikacin and meropenem), administered according to the antibiogram. Anticoagulant treatment with low molecular weight heparin (1.7 mg/kg, twice daily) was also initiated due to the prothrombotic state.

At months 2 of life, she underwent surgical revision with resection of the small intestine (evidence on biopsy of covered perforations and areas of stenosis secondary to the previous ischemic event), and termino-terminal ileo-ileal anastomosis (the total remaining length of small intestine, distal to the jejunostomy, was 35 cm). For the first month of life, a total parenteral nutrition was given, in order to support the enteral feeding, performed at first with an amino acid-based formula, administered though nasogastric tube for around two weeks, and then changed with a standard starting milk formula. The subsequent clinical course was uneventful until five months of age. At that time, she developed cholestatic jaundice (total bilirubin 11.5 mg/dL, with direct bilirubin levels of 6.44 mg/dL), for which oral treatment with ursodeoxycholic acid (30 mg/kg/day, divided into 3 doses) was initiated, along with supplementation of fat-soluble vitamins (vitamin K at 100 mcg/day and vitamin D at 800 IU/day) and medium-chain triglycerides (MCT) (1 mL/kg per feeding, 6 feedings per day). She then began exclusive enteral nutrition including complementary feeding. However, due to persistent feeding difficulties (caused by weak suction and poor coordination of swallowing), including problems with solid food, as well as diarrhea leading to poor weight gain (4930 g, <0.4th percentile, −2.86 SD), a special low-lactose formula was introduced. This finally proved beneficial, allowing tolerance and slow but steady growth. She was discharged at 6 months of age and enrolled in a multidisciplinary follow-up program including auxology, gastroenterology/surgery, neurodevelopment, ophthalmology, and audiology. At the first follow-up evaluation, approximately one week after discharge, worsening cholestasis was observed, with direct hyperbilirubinemia (11.5 mg/dL) and indirect hyperbilirubinemia (6.44 mg/dL), associated with transient acholic stools and coagulation abnormalities (INR 2.3, normal values 0.8–1.2). The coagulation disorder was successfully treated with intramuscular vitamin K (1 mg/kg). Abdominal ultrasound showed dilation of the intrahepatic and extrahepatic bile ducts, including the common bile duct (4 mm diameter, normal value 1 mm) [6], along with a hyperechogenic suspension observed within the gallbladder and common bile duct.

At 8 months of age, laboratory and ultrasound findings of cholestasis persisted, associated with signs of portal hypertension, including splenomegaly (see below) and thrombocytopenia (PLT count around 100,000/μL), for which esophagogastroduodenoscopy was recommended. The related deficiency of fat-soluble vitamins was still treated with oral administration of vitamins K and D (see above). Due to persistent poor growth, reintroduction of cow’s milk proteins and maltodextrin, along with MCT supplementation, was recommended. In the following months, our infant showed gradual, although minimal, growth and clinical improvement. At present, aged 1 year, 11 months, and 12 days, her anthropometric measures are as follows: weight 10 kg (12th percentile; −1.16 SD), length 82 cm (12th percentile; −1.19 SD), and OFC 42.5 cm (<0.4th percentile; −3.36 SD), according to World Health Organization growth charts [7]. Cholestasis has presently resolved, and therapy with ursodeoxycholic acid and vitamin K has been discontinued. She continues to receive vitamin D due to ongoing deficiency, as documented by low plasma levels (12.3 ng/mL, n.v. 32–100). Further blood tests reveal normal coagulation and liver function indices, as well as normal blood counts (thrombocytopenia is currently absent). Abdominal ultrasound shows a normal gallbladder and hepatic vascular flow; however, the liver exhibits irregular margins and splenomegaly (maximum splenic length 9.4 cm, compared to 6 cm as the upper normal limit for age-matched healthy subjects). The splenic width was assessed at the hilum on a longitudinal coronal image, according to the American Society for Pediatric Radiology recommendations [8].

At present, no other clinical or ultrasound signs of portal hypertension are noted; therefore, postponement of the esophagogastroduodenoscopy was deemed appropriate. She is currently on a free diet and is also followed by child neuropsychiatrists. At their last evaluation, performed at 2 years of age, she was noted to have microcephaly with a head circumference of 43 cm (<3rd percentile; −3.1 SD). The patient is alert and shows visual engagement, a social smile, and responds to her name. She exhibits adequate reciprocity and maintains attention during clinician-led activities. Mimic-gestural communication is present, along with deictic and proto-declarative gestures. Expressive language is underdeveloped and limited to a few words; sentence structure has not yet emerged. Verbal comprehension is intact. During the evaluation, she did not build a tower and was unable to complete shape-sorting games. Osteotendinous reflexes were normally elicited in all four limbs. Muscle tone and trophism, as well as walking ability, are normal. Up to approximately 18 months of age, cranial and suture ultrasonography were effective in monitoring and excluding hydrocephalus or other abnormalities potentially associated with unilateral coronal craniosynostosis. Given the persistence of microcephaly, and considering the limited diagnostic yield of transfontanellar ultrasound at this stage, a 3D cranial computed tomography scan—so far deferred due to the absence of neurological or ophthalmological signs indicative of intracranial hypertension, and in order to avoid both unnecessary and untimely exposure to ionizing radiation—is now planned. A neuropsychiatric follow-up (and, if necessary, neurosurgical evaluation) has also been scheduled to monitor psychomotor developmental milestones. In addition, speech and psychomotor therapy have been prescribed to enhance expressive vocabulary, foster sentence construction, consolidate and complete the phonetic–phonological repertoire, and improve verbal decoding, attentional control, executive functioning, working memory, visuomotor coordination, and visuoperceptual abilities (Supplementary File S1).

## 3. Discussion and Literature Review

CFNS, a subtype of craniofrontonasal dysplasia, is characterized in females by coronal craniosynostosis, frizzy hair, frontal bossing, craniofacial asymmetry, and distinctive facial dysmorphisms (i.e., hypertelorism, downslanting palpebral fissures, broad bifid nose, and a low posterior hairline with an anterior widow’s peak). Common extracranial features include sloping shoulders with dysplastic clavicles, mild cutaneous syndactyly, and characteristic longitudinal splitting of the nails. Additional anomalies such as cleft lip and palate, duplication of the first digit, congenital diaphragmatic hernia, and agenesis of the *corpus callosum* may also be observed [9,10]. Males described to date are fewer and usually mildly affected, typically showing only hypertelorism and, more rarely, cleft lip or palate.

The CFNS *locus* is located in the centromeric region of the X chromosome (Xq12), where the *EFNB1* gene is found. Due to the range of malformations observed in *Efnb1*-deficient mice and the unusual inheritance pattern resembling CFNS [11], Wieland et al. [12] investigated the role of *EFNB1* in three families affected by CFNS. In one family, a deletion involving exons 2–5 was identified, while the other two harbored missense variants. Both of these alterations were located in the multimerization and receptor-interaction domains of the extracellular portion of the encoded protein, ephrin-B1. Ephrin-B1 is involved in cell signaling and plays a crucial role in the proper development of the nervous and vascular systems. Specifically, it interacts with Eph receptors, and this interaction is essential for the formation of facial and cranial structures during embryonic development [13]. Beyond its role in craniofacial morphogenesis, *EFNB1* is also critically involved in vascular development. Ephrin-B1/Eph receptor interactions regulate endothelial cell migration, vessel sprouting, and vascular patterning during embryogenesis—processes essential for proper angiogenesis and tissue organization [14]. Disruption of this signaling pathway may therefore underlie not only the craniofacial manifestations of CFNS but also the extracranial anomalies occasionally reported in affected patients. A deeper understanding of the vascular functions of *EFNB1* provides a broader framework for interpreting the clinical variability observed in CFNS, including the present case, characterized by superior mesenteric artery thrombosis.

CFNS is an unusual X-linked condition, as it predominantly affects females. This contrasts with most X-linked disorders, which typically affect males or are more severe in them [15]. One explanation for this discrepancy lies in the so-called “cellular interference” phenomenon. While *EFNB1* was once considered redundant or non-essential, some males with a complete loss of *EFNB1* show no clinical symptoms, suggesting the gene may be dispensable in certain contexts. However, a small number of males have been reported as mosaics for *EFNB1* mutations, meaning the mutation arose post-zygotically and is present only in some tissues. A recent study showed that mosaic males can exhibit clinical features resembling those seen in affected females. It is believed that the phenotypic expression of CFNS is related to varying levels of *EFNB1* expression in different tissues. In both affected females and mosaic males, the presence of two distinct cell populations—due to random X-chromosome inactivation in females or somatic mosaicism in males—may lead to the characteristic clinical manifestations [16].

Although our proband received a confirmed diagnosis of CFND, she presented with thrombosis of the superior mesenteric artery, a finding not typically associated with this condition. Posey et al. [17] reported that patients already diagnosed with one rare genetic disorder have approximately a 5% risk of harboring a second. A more recent study [18] confirmed that multiple genetic conditions can co-occur in 2–7.5% of diagnosed cases. To date, there is no significant evidence directly linking CFND to an increased risk of thrombosis. However, *EFNB1*—the gene mutated in CFND—is involved in cellular signaling processes crucial for vascular and nervous system development. Although CFND is primarily a craniofacial syndrome, some studies suggest that *EFNB1* alterations may affect the vascular system [19]. Specifically, Wu et al. demonstrated in a mouse model that *EFNB1*, through reverse signaling, limits the activity of RhoA, a small GTPase involved in smooth muscle contraction and cytoskeletal dynamics [14]. In *Efnb1* knockout mice, increased RhoA activity results in enhanced phosphorylation of the myosin light chain in mesenteric arteries and elevated arterial blood pressure under stress conditions, potentially predisposing to clot formation and/or thrombotic events. Moreover, a duplication involving *EFNB1*, along with *STARD8* and *PJA1*, could collectively disrupt vascular homeostasis: *EFNB1* overexpression may interfere with endothelial signaling and RhoA-mediated smooth muscle contractility; *STARD8* may further modulate RhoA activity, impacting vascular stability [20]; and *PJA1* could alter protein ubiquitination pathways critical for vascular function [21]. Together, these mechanisms may have increased thrombotic susceptibility in the present patient. Indeed, in our case, a microduplication at Xq13.1 [(67898988_68690813)×3], encompassing *EFNB1* and neighboring genes such as *STARD8* and *PJA1*, was identified in a female patient with classic CFNS features. Interestingly, the same rearrangement was detected in the proband’s father, who showed a milder phenotype—limited to isolated hypertelorism—with no other obvious clinical signs. The distinctiveness of this case lies in its association with NEC, likely secondary to superior mesenteric artery thrombosis—a rare but severe condition requiring surgery and intensive care [22]. While the thrombosis might be explained by homozygosity for the *MTHFR* A1298C polymorphism, the complex phenotype observed in our patient may result from multiple contributing factors, including additional genomic variants (e.g., intronic or regulatory changes, or alterations in yet unknown genes) and epigenetic mechanisms that modulate *EFNB1* expression, as observed in contiguous gene syndromes [23,24]. Supporting this hypothesis, Petit et al. [25] reported a case involving a male with congenital diaphragmatic hernia (CDH) and a microduplication at Xq12–q13.1, encompassing *EFNB1*. Although *EFNB1* is clearly involved in craniofacial development, the authors noted that the other duplicated genes in their patient were not directly associated with CFND. Similarly, in our case, the duplicated region includes *EFNB1*, *STARD8*, and *PJA1*. This raises an intriguing hypothesis: the co-duplication of these three genes, though not classically linked to CFND, may contribute synergistically—or as phenotypic modifiers—to a more complex clinical picture. Specifically, *STARD8*, which encodes a Rho-GTPase activating protein, may also play a role in craniofacial development through its involvement in cell adhesion at focal adhesion sites [26]. Likewise, *PJA1*, encoding an E3 ubiquitin ligase, may affect protein degradation and intracellular signaling during morphogenesis [27]. The comparison between these two cases suggests that, while not causative on their own, these duplicated genes may contribute to CFND pathogenesis or phenotypic variability, including vascular abnormalities as observed in the proband. Moreover, although thrombosis and *MTHFR* polymorphisms have been described in various clinical contexts, no direct association between CFND and mesenteric artery thrombosis linked to A1298C polymorphism has been previously documented. Therefore, this may represent the first reported case of NEC and abdominal artery thrombosis in a patient with CFND and homozygous *MTHFR* A1298C variant. Nevertheless, other contributing factors such as prematurity or sepsis [28] must be considered, given the absence of any correlation with central catheter placement (which occurred after the onset of symptoms). The patient also developed chronic liver disease, likely due to prolonged total parenteral nutrition and the onset of short bowel syndrome, resulting in both malabsorption and impaired growth.

To contextualize our findings, we conducted a mini-review of the recent literature (2004–2025) on similar neonatal cases. We searched the PubMed and Scopus databases, including only case reports and reviews in English. Letters and case series were excluded. The following keywords (alone or in combination) were used: “craniofrontonasal syndrome”, “craniofrontonasal dysplasia”, “necrotizing enterocolitis”, “gastrointestinal”, “complications”, “coagulation”, “thrombosis”, “newborns”, “pediatric”, “*EFNB1*”. A comparison between our case and those reported in the literature is provided in Table 1, highlighting demographic and perinatal data, clinical features (including any associated risk factors and/or additional findings beyond craniofacial dysmorphisms), genomic profiles, and outcomes.

All identified patients exhibit craniofacial dysmorphisms consistent with alterations in *EFNB1* or the Xq13.1 region, suggesting a shared molecular basis for disease pathogenesis. The main differences lie in the extent of neurological and systemic involvement: complex structural variants are associated with more severe phenotypes and poorer outcomes, whereas isolated point mutations tend to result in milder clinical courses and preserved development. Additional manifestations primarily involve the central nervous system (e.g., agenesis of the *corpus callosum*, cerebellar anomalies), as well as ocular conditions (e.g., pigmentary glaucoma) and skeletal abnormalities (e.g., scoliosis, hip dislocation, *cranium bifidum occultum*, and other skull defects). To date, no CFND patients with coexisting gastrointestinal anomalies or vascular defects/thrombotic conditions have been reported, aside from the aforementioned case of congenital diaphragmatic hernia (CDH).

## 4. Conclusions

The present report aims to further define the genomic and clinical characteristics of CFND, and to provide useful guidance for the accurate classification and management of affected patients. Our study also opens the possibility of exploring the role of gene mutations and polymorphisms in the pathogenesis of vascular complications in conditions such as craniofrontonasal dysplasia, and of establishing whether a pathogenic link exists between them.

## Figures and Tables

**Table 1:** Comparison between CFND cases reported in the literature and the present patient, with reference to clinical and genetic features, diagnostic workup, therapeutic approach, and outcomes.

Authors	Gestational Age	Sex	Concurrent Risk Factors/Conditions	Involved Genes	Dysmorphic Features	Other Manifestations	Outcome
Petit F et al. [25]	32	M	Congenital right diaphragmatic hernia diagnosed during the second trimester of pregnancy.	Xq12q13.1 microduplication including five contiguous genes (*OPHN1*, *YIPF6*, *STARD8, EFNB1* and *PJA1*).	Hypertelorism, epicanthic folds, bifid nasal tip, laterally interrupted eyebrows, low-set and dysplastic ears, right camptodactyly of the second and third fingers, low-set right nipple, and ectopic left testis.	The congenital diaphragmatic hernia was surgically repaired on day 4 of life. At 6 months of age, he underwent a *fundoplicatio* and gastrostomy tube placement due to severe gastroesophageal reflux.	Walking achieved at age 19 months. Speech delay, mild developmental delay.
Ibrahim I et al. [29]	39 + 4	F	Mother affected by CFND	Homozygous pathogenic variant in *EXOSC3*: C.395A > C, p.ASp132Ala, inherited from the mother. Likely pathogenic duplication at Xq13.1 (including *EFNB1*) and a paternally inherited 16p11.2 duplication, classified as VUS.	Ocular hypertelorism, broad nasal tip and bifid nose, upturned earlobes, clinodactyly, craniosynostosis, polysyndactyly, left hip dislocation, and scoliosis.	At 2 years of age, she was unable to walk independently and showed severe motor and language delays. Brain MRI revealed agenesis of the *corpus callosum*, cerebellar hypoplasia, and loss of cortical gray-white matter differentiation.	She passed away at 30 months due to respiratory complications.
Shotelersuka V et al. [30]	Full term	M	-	c.253C > T (p.Gln85Ter) in *EFNB1*.	Plagiocephaly, frontal bossing, mild widow’s peak, hypertelorism, telecanthus, orbital displacement, high-arched palate, broad nasal root, and bifid nasal tip.	Inferior vermian and right cerebellar hypoplasia seen on CT scan	Normal development.
Acosta-Fernández E et al. [31]	Full term	F	-	Heterozygous c.646G > T transversion in exon 5 of *EFNB1*.	Brachyplagiocephaly, frontal bossing, hypertelorism, facial asymmetry, and various cranial and facial abnormalities.	A CT scan revealed *cranium bifidum occultum* and other skull issues.	At age 4, diagnosed with pigmentary glaucoma. Normal intelligence, treated for ADHD.
Our patient	36 + 4	F	Mother with type 1 diabetes mellitus.	Microduplication of 791 Kb on chromosome Xq13.1 including the *EFNB1*, *STARD8*, and *PJA1* genes. The A1298C polymorphism of *MTHFR* in homozygosity.	Plagiocephaly, craniofacial asymmetry, prominent frontal drafts, epicanthus, hypertelorism, broad and bifid nasal tip, short and thick philtrum, high-arched palate, brachydactyly and clinodactyly.	NEC due to thrombosis of the superior mesenteric artery. She developed short bowel syndrome leading to malabsorption, cholestatic liver disease and growth impairment.	Global neurodevelopmental delay.

ADHD = attention deficit and hyperactivity disorder; CT = computed tomography; F = female; M = male; MRI = magnetic resonance imaging; VUS = variant of uncertain significance.

## Data Availability

Data supporting reported results are available from the corresponding author on reasonable request.

## Supplementary Materials

The following supporting information can be downloaded at: https://www.mdpi.com/article/10.3390/jcm14197055/s1, Supplementary File S1: Clinical timeline summarizing pre- and perinatal events, NEC onset, surgical interventions, initiation and discon-tinuation of anticoagulation, transitions between total parenteral nutrition (TPN) and enteral feeding, cholestasis diagnosis and treatment, and developmental assessments up to 2 years of age.

## Abbreviations

The following abbreviations are used in this manuscript:

CFND	Craniofrontonasal dysplasia
CFNS	Craniofrontonasal syndrome
CDH	Congenital diaphragmatic hernia
*EFNB1*	Ephrin-B1 gene
NEC	Necrotizing enterocolitis
n.v.	normal values
US	Ultrasound
