# Supplementary figures and images for: Necrotizing Enterocolitis Due to Mesenteric Artery Thrombosis in a Patient with Craniofrontonasal Dysplasia: Casual or Causal Association?
Source: J Clin Med. 2025 Oct 6;14(19):7055. doi: 10.3390/jcm14197055 (PMC12525834; doi:10.3390/jcm14197055)

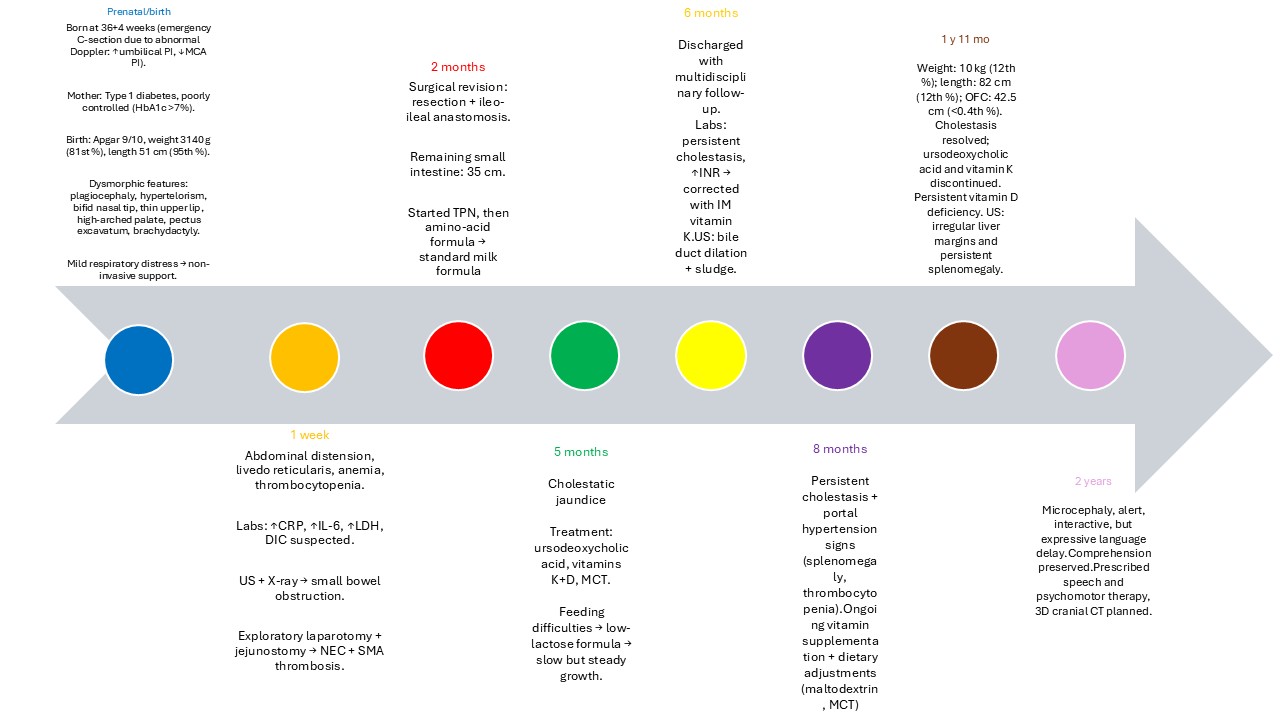

Supplement: Supplementary file 1 [file jcm-14-07055-s001.zip › jcm-3850857-supplementary.jpg]
